# Group A β-hemolytic streptococcus causing purpura fulminans: two case reports

**DOI:** 10.3389/fimmu.2026.1659910

**Published:** 2026-02-10

**Authors:** A. Alkhatatbeh, Jiechen Chen, Yongru Chen, Zebin Ma, Hongjiang Chen, Bin Chen, Yongfei Zhou, Xue Xia, Xiaoqing Huang, Shuting Hong, Jun Hu

**Affiliations:** 1 Department of Orthopedics Surgery, The First Affiliated Hospital of Shantou University Medical College, Shantou, Guangdong, China; 2 Department of Emergency Intensive Care Unit (EICU), The First Affiliated Hospital of Shantou University Medical College, Shantou, Guangdong, China

**Keywords:** case report, disseminated intravascular coagulation, multidisciplinary approach, multi-organ failure, purpura fulminans, septic shock, streptococcus pyogenes

## Abstract

Purpura fulminans (PF) is a rare, life-threatening emergency characterized by rapidly progressive purpura evolving into hemorrhagic bullae and skin necrosis due to dermal microvascular thrombosis, typically accompanied by shock and disseminated intravascular coagulation (DIC). We report two fatal adult cases of infection-associated PF caused by Streptococcus pyogenes (group A streptococcus). Both patients presented with early gastrointestinal symptoms and rapidly developed septic shock, severe coagulopathy compatible with overt DIC, and extensive lower-limb purpura with bullae and necrosis. Pathogen identification was supported by next-generation sequencing from blood and/or tissue specimens. Management required immediate resuscitation, organ support, targeted anti–group A streptococcal therapy, and surgical source control; nevertheless, both patients deteriorated with refractory multiorgan failure. These cases highlight that gastrointestinal symptoms may precede fulminant invasive S. pyogenes infection with PF and streptococcal toxic shock syndrome overlap. Early recognition of the clinical pattern (shock + rapidly progressive purpura/bullae + DIC) and aggressive multidisciplinary management are essential.

## Introduction

Purpura fulminans (PF) is a rare but life-threatening dermatologic and hematologic emergency characterized by the acute onset of rapidly progressive purpura that evolves into hemorrhagic bullae and skin necrosis due to microvascular thrombosis, typically accompanied by disseminated intravascular coagulation (DIC), shock, and multi-organ failure. It is often associated with severe infections, primarily caused by pathogens like Neisseria meningitidis and Streptococcus pneumoniae (1). In infection-associated PF, key diagnostic features include an acute infectious event with rapidly progressive DIC (thrombocytopenia, prolonged prothrombin time, low fibrinogen level, and elevated D-dimer), lactic acidosis disproportionate to the degree of hemodynamic shock, and extensive skin mottling that progresses to a purpuric rash (1). These findings should prompt immediate recognition and aggressive management because progression can be rapid and catastrophic.

PF and STSS are related but distinct entities. STSS is a toxin-mediated invasive Streptococcus pyogenes (group A streptococcus, GAS) infection defined by the CDC/CSTE 2010 criteria (2) as: (A) hypotension (SBP ≤90 mmHg in adults), (B) at least two organ-system criteria (renal impairment, coagulopathy/DIC, liver involvement, ARDS/capillary leak, generalized erythematous rash, or soft-tissue necrosis), and (C) microbiologic evidence of GAS (sterile-site isolation = confirmed; non-sterile site = probable). Both patients fulfilled the clinical criteria for STSS, and GAS was identified by next-generation sequencing from blood and/or tissue specimens; therefore, an STSS overlap phenotype was considered.

PF can be classified into three main types: (A) Neonatal PF (1:1,000,000 live births), which is caused by homozygous or compound heterozygous loss of PROC or, less commonly, PROS1. (B) Idiopathic PF, which is extremely rare and linked to autoantibodies against protein S. It has also been reported in patients on anti-cancer immunotherapy due to immune dysregulation. (C) Infection-associated PF, the most common form, is triggered by bacterial infections (1). PF due to S. pyogenes is a rare cause of the disease in adults; however, when it occurs, it may progress to streptococcal toxic shock syndrome (STSS), septic shock, and DIC. The condition is difficult to diagnose early as the initial symptoms are non-specific and may include fever, malaise, and localized skin changes (3). These initial symptoms rapidly progress to severe manifestations like widespread purpura, skin necrosis, and organ failure.

The pathophysiology of PF is the formation of microthrombi that block dermal capillaries leading to hemorrhagic infarction. As ischemia and vascular damage progress, blood components are extravasated into the dermis, and petechiae form, then hemorrhagic macules and eventually necrosis of skin, fascia, and muscle. This is compounded by secondary infections that worsen the patient’s condition and contribute to increased mortality. The prognosis of PF is poor with a high mortality rate due to multi-organ failure and septic shock often exceeding 60% (1).

This paper reports two rare cases of acute infectious purpura fulminans (PF) caused by *Streptococcus pyogenes* (*S. pyogenes*), involving a 66-year-old and a 44-year-old male. Including the present cases, a total of seven adult cases of S. pyogenes–associated infection-related PF have been reported in the literature (**Table 1**; ref (4–8)), suggesting a high risk of rapid deterioration and poor outcomes; therefore, invasive S. pyogenes should be considered in adults presenting with PF and septic shock, particularly when accompanied by coagulopathy and rapidly progressive skin necrosis.

**Table 1:** Summary of reported adult cases of Streptococcus pyogenes associated infection-related purpura fulminans, including the present two cases.

Study (year)	Age/sex	Initial presentation	Microbiology	Key treatments reported	Surgery/amputation	Outcome
Ward KM et al. (2002)	62/F	Acral purpura/gangrene	Group A streptococcus	Not reported	Not reported	Died
Thomas D et al. (2009)	36/M	Vomiting/diarrhea + fever	Blood cultures: Group A streptococcus	Antibiotics (amoxicillin/gentamicin/clindamycin) + IVIG	Amputation	Survived
Ashokkumar GK et al. (2015)	72/F	Acute gastroenteritis	Blood cultures: Group A streptococcus	Antibiotics (Meropenem + clindamycin); anticoagulation	Not reported	Survived
Gupta D et al. (2016)	50/M	Fever + limb swelling	Group A streptococcus	Antibiotics (Vancomycin + piperacillin/tazobactam); fluids; platelet transfusion	Debridement; later skin grafting (left arm)	Survived
Hartmann B et al. (2025)	39/M	Fever/myalgias	Blood cultures: Group A streptococcus	Antibiotics (Vancomycin/ceftriaxone, clindamycin/meropenem, ampicillin–sulbactam); heparin; IVIG	Bilateral upper-extremity transradial amputation; bilateral above-knee amputation	Survived
Present case 1 (2020)	66/M	Diarrhea + limb pain/swelling	NGS/blood: Group A streptococcus	Antibiotics (Penicillin G, clindamycin); vasopressors; transfusion support	Left thigh amputation	Died
Present case 2 (2020)	44/M	Diarrhea/vomiting	NGS/necrosis tissue: Group A streptococcus	Antibiotics (imipenem and vancomycin); plasma transfusion	Bilateral fasciotomy (lower limb)	Died

F, female; M, male; GAS, group A streptococcus (Streptococcus pyogenes); IVIG, intravenous immunoglobulin; NGS, next-generation sequencing; PF, purpura fulminans.

## Case presentation 1

**Figure 1:**
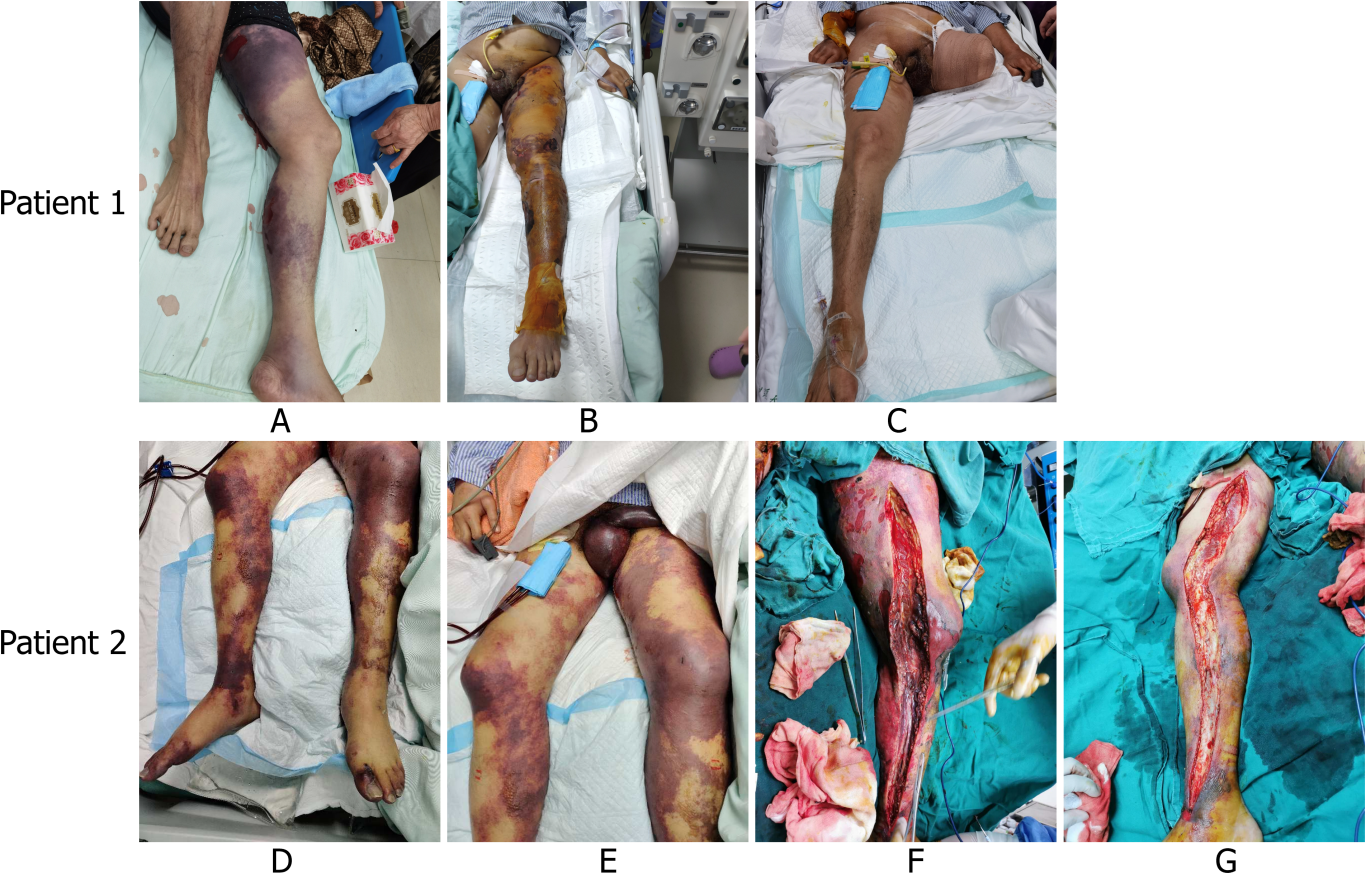
Representative clinical photographs demonstrating the rapid evolution of infection-associated purpura fulminans (PF), highlighting the teaching point that progression can be swift and should prompt urgent recognition and escalation of care. **(A)** Patient 1 at admission: purpura on the left lower limb. **(B)** Patient 1: progression/worsening of purpura. **(C)** Patient 1: post–left thigh amputation. **(D, E)** Patient 2: purpura on both lower limbs at presentation. **(F, G)** Patient 2: postoperative findings after bilateral lower-limb fasciotomy and debridement.

A 66-year-old male with a history of gouty arthritis and prior sigmoid colon cancer surgery was admitted to the Emergency Intensive Care Unit on Day 0, after experiencing two days of left lower limb pain and swelling, along with recurrent diarrhea and shortness of breath. Upon arrival, he was in severe hypotension (78/30 mmHg), with a pulse of 120 bpm, a respiratory rate of 25 bpm, and a temperature of 37.0 °C. Physical examination revealed dark purplish-red patches, blood blisters, and areas of skin detachment on his left lower limb, extending to the scrotum and abdomen, raising concerns for sepsis and purpura fulminans (PF) (**Figure 1A**).

Initial laboratory evaluation showed severe lactic acidosis, acute kidney injury, hepatic injury, and marked systemic inflammation (**Table 2**). Coagulopathy with severe thrombocytopenia and markedly elevated D-dimer was present; using the ISTH overt DIC scoring system, the findings were compatible with overt DIC. Blood cultures and pustule fluid samples were sent for next-generation sequencing (NGS), which identified Streptococcus pyogenes.

**Table 2:** Laboratory data comparison of two cases.

Variable	Reference Range (Adults)	Patient 1	Patient 2
Renal			
Creatinine (µmol/L)	45-133	207	256
Metabolic			
Lactic acid (mmol/L)	0.5-1.7	11.33	>20.00
Hepatic/tissue injury			
Total bilirubin (µmol/L)	0.00-26.00	26.36	246.15
ALT (U/L)	7-40	170.09	519.05
AST (U/L)	13-35	757.39	995.50
LDH (U/L)	120-250	1866	2161
Creatine Kinase (U/L)	40-200	342.3	771.20
Cardiac			
High-sensitivity troponin I (pg/mL)	0-34.2	323.50	403.00
Inflammatory			
White cell count (×10^^9^/L)	3.50-9.50	24.81	17.54
Neutrophils (%)	40%-75%	97.27	92.84
CRP (mg/L)	0-8.0	345	154
PCT (ng/mL)	0-0.04	>150.00	81.6
Hematology			
Hemoglobin (g/L)	130-175	65	94
Coagulation			
Platelet count (×10^^9^/L)	125-350	14	18
INR	0.80-1.15	1.25	2.16
Activated Partial Thromboplastin Time (sec)	23.30-32.50	65.7	51.3
D-dimer (µg/L FEU)	<550.00	8000	40330
Fibrinogen (g/L)	1.80-3.50	4.97	1.25

ALT, alanine aminotransferase; AST, aspartate aminotransferase; APTT, activated partial thromboplastin time; CK, creatine kinase; INR, International normalized ratio; CRP, C-reactive protein; LDH, lactate dehydrogenase; PCT, procalcitonin; F, female; M, male.

Given his severe clinical state, he was started on targeted anti–group A streptococcal therapy (penicillin G plus clindamycin, the latter to inhibit toxin production). Fluid resuscitation was initiated for hypotension, and norepinephrine was used for vasopressor support. He required mechanical ventilation for respiratory distress and continuous renal replacement therapy (CRRT) due to acute kidney failure.

Despite aggressive treatment, the purpura continued to spread, and the necrosis worsened (**Figure 1B**). After evaluation by a multidisciplinary team, a left thigh amputation was performed on Day 6, in an effort to control the infection (**Figure 1C**). Following surgery, his condition initially improved, his vital signs stabilized, and he regained consciousness. By Day 13, his endotracheal tube was removed.

However, on Day 15, he developed severe respiratory distress due to sputum obstruction, requiring reintubation. A sputum culture identified Aspergillus fumigatus, indicating a hospital-acquired fungal infection. Despite antifungal therapy with voriconazole, his condition deteriorated. On Day 30, he suffered multi-organ failure and, despite resuscitation efforts, passed away.

## Case presentation 2

A 44-year-old male patient presented to the emergency department on Day 0, with recurrent diarrhea, vomiting, and mottled skin changes, accompanied by ecchymosis on both lower limbs over the past two days. Physical examination revealed a normal body temperature, but tachycardia (heart rate 120 bpm) and hypotension (blood pressure 82/43 mmHg) were also noted. The skin showed mottled changes, with large ecchymoses on both lower limbs, primarily around the knees. Multiple tense bullae of varying sizes were scattered on the inner thighs, and there was skin breakdown on the first toe and sole of the left foot without significant bleeding or purulent discharge. The extremities were cold and clammy, with normal muscle strength and tone. The patient reported that two days prior, he had developed a skin lesion on the sole of his left foot, which he treated with a non-sterilized blade. He had no known chronic illnesses, was not taking regular medications (including anticoagulants or immunosuppressants), and had no relevant family history.

Initial laboratory results (**Table 2**) showed leukocytosis with neutrophilia, severe lactic acidosis, and marked coagulopathy (severe thrombocytopenia, prolonged coagulation times, and markedly elevated D-dimer), consistent with sepsis-associated coagulopathy and overt DIC in the appropriate clinical context. Based on the rapidly progressive purpura/bullae with shock and coagulopathy, infection-associated purpura fulminans was considered.

Within 1 hour of admission, the patient developed hyperpyrexia (40°C) and refractory hypotension consistent with septic shock and rapidly progressed to cardiac arrest. Cardiopulmonary resuscitation was initiated immediately with intravenous epinephrine; the patient was intubated and mechanically ventilated. Return of spontaneous circulation was achieved, and he was treated with broad empiric antibiotics (imipenem and vancomycin), plasma transfusion for coagulopathy, anti-shock therapy, and renal replacement therapy.

After treatment, infection markers showed a decline, but tension in the lower limbs increased, ecchymosis worsened, and skin necrosis with a foul odor developed (**Figure 1D** and **Figure 1E**). On Day 5, bilateral lower limb fasciotomy was performed at the bedside (**Figure 1F** and **Figure 1G**), and tissue samples were sent for next-generation sequencing (NGS). On Day 8, NGS results confirmed that the purulent fluid was from a Streptococcus pyogenes infection.

On Day 15, the patient experienced progressive hypotension and cardiac arrest. Immediate chest compressions and intravenous epinephrine were administered, but spontaneous circulation could not be restored. Despite 30 minutes of aggressive resuscitation, he was declared clinically dead.

## Discussion

Purpura fulminans (PF) is a rare but life-threatening condition characterized by rapidly progressive purpura that can evolve into hemorrhagic bullae and skin necrosis in the setting of severe infection and dysregulated coagulation. Reported mortality is high, and survivors often have major tissue loss and amputations (1, 9). In many patients, clinical deterioration is driven by microvascular thrombosis and systemic coagulopathy rather than uncontrolled local infection alone (9). PF is commonly categorized into neonatal, idiopathic, and infection-associated forms, with infection-associated purpura fulminans (IAPF) being the most frequent presentation (1, 9). IAPF is more commonly reported in children and is most often linked to pathogens such as Neisseria meningitidis and Streptococcus pneumoniae, although other bacteria, viruses, and protozoa have also been implicated (9, 10). In contrast, the present report describes adult IAPF caused by invasive Streptococcus pyogenes, which is uncommon but clinically important because of its rapid progression and association with streptococcal toxic shock syndrome (STSS) (3, 8).

Although PF and STSS are distinct entities, they frequently overlap in invasive group A streptococcal infection, where toxin-mediated shock and endothelial injury can amplify coagulopathy and tissue necrosis (3, 8, 9). In our two patients, the combination of confirmed group A streptococcus, refractory hypotension requiring vasopressors, and multiorgan dysfunction supported an STSS overlap phenotype, which is consistent with prior adult reports (3, 8). This overlap is clinically important because it should heighten urgency for early source control, toxin suppressive antibiotics, and aggressive organ support.

The laboratory profile in both cases was consistent with severe sepsis-associated coagulopathy progressing to overt disseminated intravascular coagulation (DIC), with profound thrombocytopenia, markedly elevated D-dimer, prolonged coagulation parameters, and evolving multiorgan failure (1, 9). Using the ISTH overt DIC framework, and considering INR as a practical surrogate for prothrombin time prolongation when only INR is available, both cases were compatible with overt DIC in the appropriate clinical context (1, 9). This criteria-based approach addresses an important diagnostic requirement and supports the diagnosis of IAPF as a clinicopathologic syndrome driven by infection, shock, and dysregulated hemostasis (1, 9).

A key clinical observation in our series is that both patients initially presented with gastrointestinal symptoms, particularly diarrhea, before rapidly developing shock and extensive purpura. Notably, gastrointestinal symptoms were also described as early features in several previously reported adult cases, including vomiting and diarrhea in Thomas et al. and acute gastroenteritis in Ashokkumar et al. (5, 6). Taken together, the adult literature summarized in **Table 1** suggests that gastrointestinal symptoms may be an early manifestation of invasive S. pyogenes infection that precedes the classic skin findings of PF in a subset of patients (**Table 1**) (4–8). This association should be interpreted cautiously, but it highlights a practical emergency medicine point: in patients with severe systemic toxicity and evolving purpura, clinicians should promptly consider IAPF with invasive group A streptococcal infection even when initial symptoms are gastrointestinal.

**Table 1** summarizes the currently reported adult cases of S. pyogenes associated infection-related PF, including the present two cases, for a total of seven adult cases (4–8). Outcomes across these cases emphasize both the severity and the variability of clinical course. Several patients survived with aggressive critical care, antibiotics, and early surgical management, but fatal outcomes were also frequent (4–8). Limb loss occurred in multiple survivors, reflecting the destructive vascular and soft tissue consequences of PF and severe streptococcal infection (4, 5, 8). These patterns support the clinical message that adult IAPF due to S. pyogenes is associated with rapid deterioration and a high risk of death or major disability, and should not be overlooked.

Management of IAPF requires immediate resuscitation, hemodynamic support, and pathogen-directed therapy, alongside close attention to coagulopathy and early involvement of surgical teams when soft tissue necrosis is suspected (1, 11–13). In invasive S. pyogenes infection, penicillin combined with clindamycin is commonly recommended because clindamycin may suppress toxin production and is less affected by high inoculum conditions (3, 9, 12). Bendapudi and Losman emphasize early transfusion support and consideration of supplementing physiologic anticoagulants such as protein C concentrate and vitamin K in selected cases, while highlighting that amputation should generally be delayed until demarcation is clear, except when wet gangrene or uncontrolled necrotic infection mandates urgent intervention (1). In our first case, amputation was performed because of rapidly progressive necrosis with concern for ongoing source and tissue nonviability in the setting of refractory shock, which aligns with the exception described for severe necrotic infection where earlier surgery may be necessary (1). Consistent with Koch et al., we also used a multidisciplinary approach involving intensive care, orthopedic surgery, and burn and wound expertise, as early surgical evaluation and coordinated supportive care may help preserve functional tissue when feasible (11).

Adjunctive therapies such as IVIG and protein C concentrate have been used in some reports of severe invasive streptococcal disease and infection-associated PF, and IVIG was administered in several published adult cases summarized in **Table 1** (5, 8, 12). In our two cases, rapid progression to refractory shock and multiorgan failure limited the feasibility and timing of additional adjunctive therapies, and management focused on immediate organ support, transfusion based correction of coagulopathy, antimicrobial therapy, and urgent surgical source control when indicated (1, 9). In addition, institutional access and emergency availability of protein C concentrate can vary across centers, which may constrain its use in time critical presentations (1). CDC guidance notes IVIG can be considered for severely ill patients early in the clinical course, although efficacy has not been proven; in our cases, the extremely rapid deterioration and timing constraints limited feasibility of adjunctive administration (12).

Finally, potential portals of entry should be considered. In Case 2, the history of a foot lesion treated with a non-sterilized blade may have provided a portal for invasive group A streptococcal infection. Recognizing such exposures may aid early suspicion of invasive streptococcal disease when shock and rapidly progressive skin findings emerge.

Despite aggressive resuscitation, mechanical ventilation, vasopressor support, renal replacement therapy, transfusion support, and surgical interventions, both patients died of rapidly progressive multiorgan failure. These outcomes underscore the fulminant nature of adult IAPF due to S. pyogenes and support the need for early recognition of the clinical trial of shock, rapidly evolving purpura with bullae or necrosis, and criteria-consistent coagulopathy, followed by immediate multidisciplinary management (1, 3, 9, 11).

## Conclusion

Purpura fulminans caused by invasive Streptococcus pyogenes is rare in adults but can progress rapidly from nonspecific symptoms, including gastrointestinal complaints, to septic shock, overt coagulopathy, and extensive skin necrosis. These two fatal cases highlight the importance of early recognition of the clinical pattern of shock with rapidly evolving purpura or hemorrhagic bullae and prompt multidisciplinary management, including targeted anti group A streptococcal therapy, aggressive organ support, and timely surgical evaluation for source control. Clinicians should maintain a high index of suspicion for invasive S. pyogenes in adults with severe systemic toxicity and rapidly progressive skin findings, because delayed diagnosis and treatment may further worsen outcomes.

## Data Availability

The original contributions presented in the study are included in the article/supplementary material. Further inquiries can be directed to the corresponding author/s.

## References

[1] Bendapudi PK Losman JA . How I diagnose and treat acute infection–associated purpura fulminans. Blood. (2025) 145:1358–68. doi: 10.1182/blood.2024025078, PMID: 39786416

[2] Centers for Disease Control and Prevention (CDC) . Streptococcal Toxic Shock Syndrome (STSS) (Streptococcus pyogenes): 2010 Case Definition (CSTE Position Statement 09-ID-60). Atlanta, GA, United States: National Notifiable Diseases Surveillance System (NNDSS). (2021). Available online at: https://ndc.services.cdc.gov/case-definitions/streptococcal-toxic-shock-syndrome-2010/.

[3] Okuzono S Ishimura M Kanno S Sonoda M Kaku N Motomura Y . Streptococcus pyogenes-purpura fulminans as an invasive form of group A streptococcal infection. Ann Clin Microbiol Antimicrob. (2018) 17:31. doi: 10.1186/s12941-018-0282-9, PMID: 29986727 PMC6036671

[4] Ward KM Celebi JT Gmyrek R Grossman ME . Acute infectious purpura fulminans associated with asplenism or hyposplenism. J Am Acad Dermatol. (2002) 47:493–6. doi: 10.1067/mjd.2002.124701, PMID: 12271289

[5] Thomas D Perpoint T Dauwalder O Lina G Floccard B Richard JC . *In vivo* and *in vitro* detection of a superantigenic toxin Vbeta signature in two forms of streptococcal toxic shock syndrome. Eur J Clin Microbiol Infect Dis. (2009) 28:671–6. doi: 10.1007/s10096-008-0671-7, PMID: 19020908

[6] Ashokkumar G Madhu R Shaji M Singh B . Bilateral symmetrical digital gangrene of upper and lower limbs due to purpura fulminans caused by *Streptococcus pyogenes:* A rare entity. Indian J Crit Care Med. (2015) 19:290–1. doi: 10.4103/0972-5229.156493, PMID: 25983440 PMC4430752

[7] Gupta D Chandrashekar L Srinivas B Thappa D . Acute infectious purpura fulminans caused by group A β-hemolytic Streptococcus: An uncommon organism. Indian Dermatol Online J. (2016) 7:132. doi: 10.4103/2229-5178.178093, PMID: 27057503 PMC4804589

[8] Hartmann B Kemper K Blaise B . Purpura fulminans secondary to toxic shock syndrome induced by Group A Streptococcal bacteremia in an adult patient. Dermatol Online J. (2025) 31. doi: 10.5070/D331365368, PMID: 40991475

[9] Colling ME Bendapudi PK . Purpura fulminans: mechanism and management of dysregulated hemostasis. Transfusion Med Rev. (2018) 32:69–76. doi: 10.1016/j.tmrv.2017.10.001, PMID: 29157918

[10] Temnithikul B Rungrunanghiranya S Limtanyakul P Angkananard T Wessagowit V . Klebsiella-induced acute infectious purpura fulminans in a thai woman: case report and review of literature. Skin Health Dis. (2023) 3:ski2.186. doi: 10.1002/ski2.186, PMID: 37275425 PMC10233087

[11] Koch C Taeger C Geis S Lonic D Heidekrueger P Dolderer J . Early fasciotomies and plastic-surgical reconstruction may enhance preservation of functional extremity length in purpura fulminans. Clin Hemorheol Microcirc. (2020) 75:267–78. doi: 10.3233/CH-190588, PMID: 31524150

[12] Centers for Disease Control and Prevention (CDC) . Clinical guidance for streptococcal toxic shock syndrome. Atlanta, GA, United States: Group A Strep. (2025). Available online at: https://www.cdc.gov/group-a-strep/hcp/clinical-guidance/streptococcal-toxic-shock-syndrome.html.

[13] World Health Organization International Committee of the Red Cross . WHO-ICRC Basic Emergency Care: approach to the acutely ill and injured. Manual. 30 October 2018. Available online at: https://www.who.int/publications/i/item/9789241513081 (Accessed January 14, 2026).

